# Adherence to dietary guidelines and dental caries among children: a longitudinal cohort study

**DOI:** 10.1093/eurpub/ckad097

**Published:** 2023-06-22

**Authors:** Agatha W van Meijeren-van Lunteren, Trudy Voortman, Eppo B Wolvius, Lea Kragt

**Affiliations:** The Generation R Study Group, Erasmus MC University Medical Center, Rotterdam, The Netherlands; Department of Oral and Maxillofacial Surgery, Special Dental Care and Orthodontics, Erasmus MC University Medical Center, Rotterdam, The Netherlands; The Generation R Study Group, Erasmus MC University Medical Center, Rotterdam, The Netherlands; Department of Epidemiology, Erasmus MC University Medical Center, Rotterdam, The Netherlands; The Generation R Study Group, Erasmus MC University Medical Center, Rotterdam, The Netherlands; Department of Oral and Maxillofacial Surgery, Special Dental Care and Orthodontics, Erasmus MC University Medical Center, Rotterdam, The Netherlands; The Generation R Study Group, Erasmus MC University Medical Center, Rotterdam, The Netherlands; Department of Oral and Maxillofacial Surgery, Special Dental Care and Orthodontics, Erasmus MC University Medical Center, Rotterdam, The Netherlands

## Abstract

**Background:**

Even though dietary sugars are the most important nutrient for caries development, the disease process is dependent on other dietary practices. The intake of individual nutrient components cannot be evaluated separately from the overall diet which includes other nutrients, foods and habits. Therefore, the aim of this study was to investigate the association between adherence to dietary guidelines and dental caries.

**Methods:**

This study was embedded in the Generation R Study, conducted in Rotterdam, the Netherlands. In total, 2911 children were included in the present analyses. Dietary intake at the age of 8 years was assessed using food-frequency questionnaires. Diet quality scores were estimated, reflecting adherence to Dutch dietary guidelines. Dental caries was assessed at the age of 13 years using intra-oral photographs. Associations were estimated using multinomial logistic regression analyses, adjusted for sociodemographic characteristics and oral hygiene practices.

**Results:**

The prevalence of dental caries at the age of 13 years was 33% (*n* = 969). Better diet quality was associated with a lower occurrence of severe dental caries after adjustments for sociodemographic factors [e.g. highest vs. lowest quartile of diet quality: odds ratio (OR) 0.62, 95% confidence interval (CI) 0.39–0.98]. After additional adjustments for oral hygiene practices, this association was not statistically significant (OR 0.65, 95% CI 0.41–1.03).

**Conclusion:**

Adherence to dietary guidelines has the potential to reduce dental caries in children; however, with proper oral hygiene practices, this relationship might be attenuated. To understand the role of dietary patterns and dental caries, the contributing role of daily eating occasions needs to be studied further.

## Introduction

Dental caries is one of the most common chronic diseases during childhood and has a negative impact on future dental and general health.^1^ Dental caries progresses with age and given its irreversibility the consequences of dental caries at a young age are lifelong, leading to high societal costs and severe impacts on quality of life.^2^ Therefore, it is important to perform research on potential determinants that could reduce the risk of caries in permanent teeth of children as early as possible.

The consumption of carbohydrates in the form of free sugars is the major risk factor for the development of dental caries,^3^ and many studies have shown a higher risk of dental caries when sugar intake increases,^4^ or when it is higher than 10% of the total daily energy intake.^5^ However, in nutritional epidemiology, the carbohydrate intake of individuals cannot be seen separated from the overall diet which includes other nutrients, foods and habits. The combination of food products, certain dietary practices or the frequency of food intake may be of importance in the development of dental caries as well.^3^^,^^6–8^

At global and national levels, nutritional guidelines advice about health favourable diets. In the Netherlands, the guidelines for a healthy diet for adults and children are developed by the Dutch Health council.^9^^,^^10^ Sugar intake and frequency of eating are not specifically included in the guideline.

Only a few studies investigated the effect of adhering to dietary guidelines on the risk of caries. Previous research from the USA showed that children who do adhere the most to the Healthy Eating Index (HEI) have a 44% lower risk for developing early childhood caries in the primary dentition than children who adhere the least to the HEI, although the HEI is not specifically developed for the prevention of dental caries.^11^ Another study among adults in the USA showed that an increase in diet quality based on the Alternative Healthy Eating Index-2010 decreased the risk of dental caries among immigrants, but not among natives.^12^

Thus, only a few studies studied the role of a whole diet in the development of dental caries, and no study has been performed among children with dental caries in the permanent dentition as the outcome of interest. Therefore, the aim of this study was to investigate the association between adherence to the Dutch dietary guidelines and dental caries among children.

## Methods

This study is conducted within the Generation R Study, which is an ongoing population-based prospective cohort study from foetal life onwards, conducted in Rotterdam, the Netherlands. The study was approved by the Medical Ethical Committee of the Erasmus Medical Centre (MEC 198.782.2001.31; MEC 2015-749-NL55105.078.15), Rotterdam, the Netherlands, and conducted according to the World Medical Association Declaration of Helsinki. All examinations, interviews and questionnaires were carried out after obtaining written informed consent of participants and their parent(s) or legal guardian(s).

**Figure 1:**
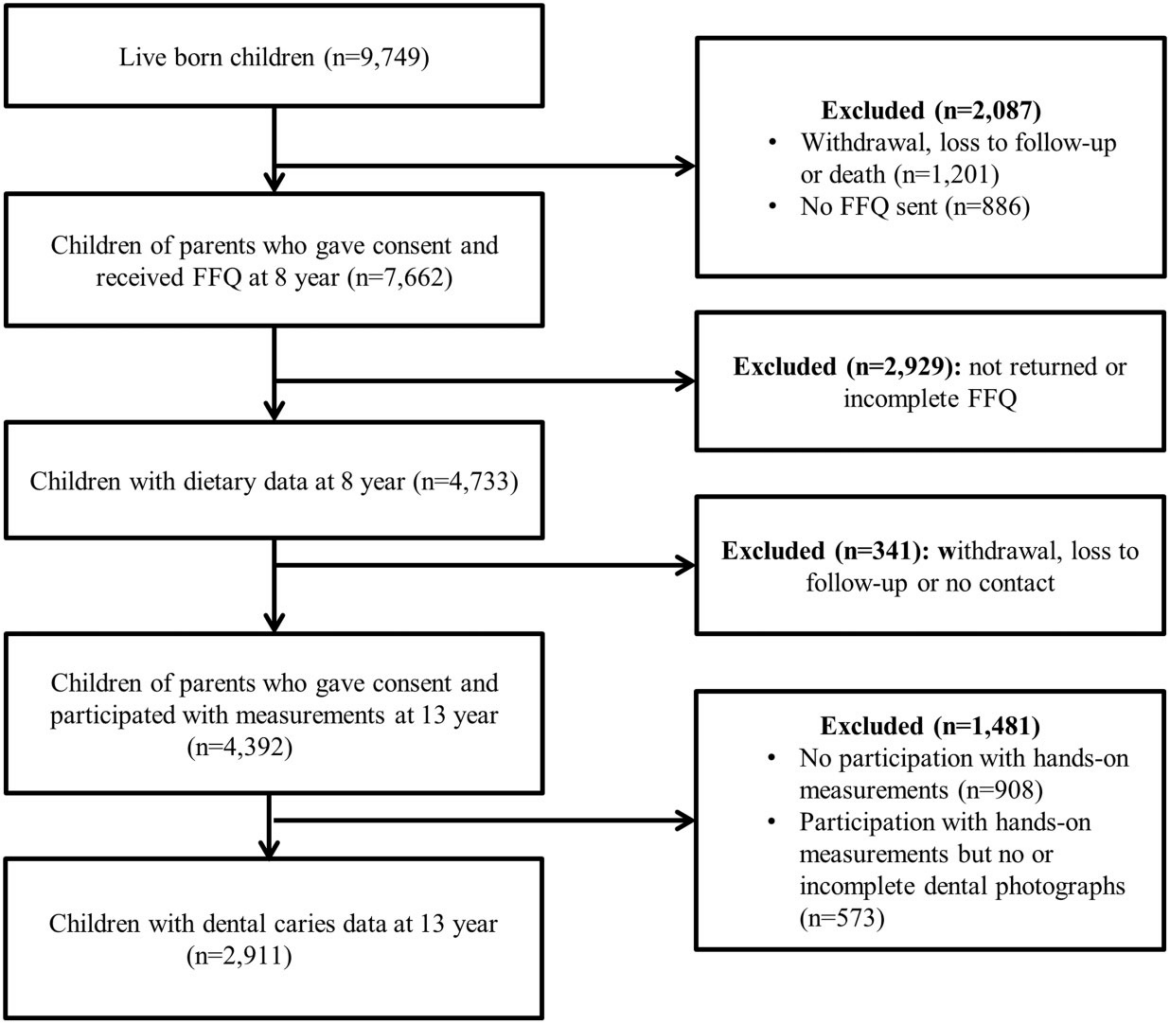
Flowchart of the study population selection

The Generation R Study is multi-disciplinary and focuses on several health outcomes from early life onwards. Women with a due date between April 2002 and January 2006 and who were registered inhabitants in the municipality of Rotterdam at the time of delivery were eligible to participate in the Generation R Study. In total, 9778 mothers were enrolled at the start of the study and gave birth to 9749 live-born children. Data collection started during pregnancy and continued prenatally and is still ongoing at various time points through different data collection methods.^13^ For the current study, data collection took place around the age of 8 years (2010–14) and around the age of 13 years (2016–19). Dietary intake was assessed in 4733 children at the age of 8 years. Of the children with available dietary intake information, 2911 children had available data on dental caries at the age of 13 years (shown in figure 1).^13^

### Adherence to dietary guidelines

Dietary intake was assessed at the age of 8 years using a validated semi-quantitative food-frequency questionnaire (FFQ),^14^^,^^15^ which was developed earlier based on the results of a national food consumption survey.^16^ The FFQ was completed by the parents of the child, using the last month as a reference. Questions concerned the frequency of consumption and portion sizes. Nutrient intakes from foods were calculated using data from the Dutch Food Composition Table (NEVO 2001).^14^^,^^15^

A food-based diet quality score, which reflects the level of adherence to dietary guidelines, was constructed based on dietary recommendations for children from the Netherlands Nutrition Centre.^9^ These were formulated by the Dutch Guidelines for a Healthy Diet of 2015.^10^^,^^15^ Ten components were included in the score namely: fruit (≥150 g/day), vegetables (≥150 g/day), whole grains (≥90 g/day), fish (≥60 g/week), legumes (≥84 g/week), nuts (≥15 g/day), dairy (≥300 g/day), oils and soft or liquid fats (≥30 g/day), sugar-containing beverages (≤150 g/day) and high-fat and processed meat (≤250 g/week). Each component was scored based on a calculation of the ratio of reported and recommended intake and truncated at 1.^15^^,^^17^ Scores of the individual components were summed, resulting in a total score for diet quality ranging from 0 to 10 on a continuous scale, with higher scores indicating better adherence to dietary guidelines.

### Dental caries

During a visit to the research centre for children aged 13 years, intra-oral photographs were taken by trained personnel using a quantitative light fluorescence camera [Qraycam™ Pro (Inspektor Research Systems BV)]. The whole dentition was captured in at least five white light and blue light pictures using cheek retractors after children had brushed their teeth for 2 min. The intra-oral photographs were scored for dental caries by two trained researchers. A random 10% of participants were evaluated double to calculate the intra-rater reliability (weighted kappa = 0.94) and inter-observer reliability (weighted kappa = 0.84) and both showed high agreement. Dental caries was assessed in the permanent dentition using the decayed, missing and filled teeth (DMFT) index.^18^ Decayed teeth were scored if localized enamel breakdown was visible or further breakdown reaching into dentin. Missing teeth were scored when elements were missing due to caries, verified on dental panoramic radiographs taken at the age of 9 years. The use of photographs for scoring dental caries in epidemiological studies has been validated previously.^19^

### Covariates

The following variables were used as covariates: child’s age, gender, total energy intake, family SES, ethnic background and oral hygiene practices.

Since dietary habits and dental caries are strongly socially patterned,^20^ family SES and ethnic background were considered as potential confounding factors. Indicators of family SES used in this study were maternal educational level and net household income and derived via parental questionnaires when the child was 6 and 13 years old, respectively. Educational level was defined according to Statistics Netherlands into three categories: ‘low,’ ‘middle’ and ‘high’.^21^ Net household income per month was dichotomized using the approximate mean monthly income per household of the year 2017 as cut-off point (≤€3600 and >€3600).^22^

Ethnic background was defined according to the Dutch classification of ethnic background, which was determined by parents’ place of birth.^23^ If both parents of the child were born in the Netherlands, the ethnic background was classified as ‘Dutch’. If one of the parents was born in another country than the Netherlands, the ethnic background was classified as ‘non-Dutch’.

Brushing frequency and oral hygiene may be important factors when studying the relationship between dietary intake and dental caries. Information about oral hygiene was assessed via parental questionnaires at the age of 13 years. For analyses, brushing frequency was dichotomized as ‘once or less per day’ and ‘twice or more per day’. We also created a dichotomous variable to take into account whether the child used any additional oral hygiene method, next to tooth brushing with toothpaste, which was based on a multiple-choice question on the use of other oral hygiene methods. Commonly used methods are the use of toothpicks, tooth floss or mouth rinsing.

### Data analyses

Multinomial logistic regression models were applied to study the associations between adherence to dietary guidelines and dental caries. Odds ratios (ORs) were estimated of having mild (DMFT 1–3) or severe caries (DMFT >3) compared with children with no caries (DMFT 0). First, the diet quality score was included in the model as a continuous determinant to estimate the ORs of dental caries per unit increase in the diet score, reflecting an increment in adherence to dietary guidelines. Second, the diet quality score was included in the model in quartiles with the lowest quartile, reflecting the worst adherence to dietary guidelines, as a reference category. Three models were built: the first model included the age and gender of the child; the second model was additionally adjusted for indicators of family SES (educational level of mother and net family household income) and ethnic background; and the third model was additionally adjusted for oral hygiene practices indicated by brushing frequency per day and other oral hygiene methods.

Multiple imputations were performed to account for information bias associated with missing data in the covariates.^24^ Missing values (<13.1%) of sociodemographic and lifestyle variables were multiple imputed by generating 10 independent datasets with the use of the Markov Chain Monte Carlo method. Imputations were based on all variables in the models, but the main determinants and the outcome were not imputed. Effect estimates for each imputed dataset were pooled and presented in this paper as ORs with 95% confidence intervals (CIs). Finally, a trend analysis was performed by including the dmft categories as a continuous term in the models. The statistical analyses were performed using SPSS (IBM Corp. Released 2017. IBM SPSS Statistics for Windows, version 25.0, IBM Corp., Armonk, NY).

### Supplementary analyses

It was investigated whether one component of the diet quality score was a driving factor for the observed inverse association with dental caries, by using adapted versions of the diet quality score omitting one component of the total score at a time. To observe the potential moderating effect of oral hygiene practices on the association between diet quality and dental caries, the results of model 1 and model 2 were also presented in different subgroups of brushing frequency. To observe the potential selection bias, a non-response analysis was performed to compare all 13-year-old children with and without missing data on diet quality and dental caries experience.

## Results

At the mean (SD) age of 8.1 (0.2), the mean (SD) diet quality score of the study population was 4.5 (1.2) (on a theoretical range of 0–10). At the mean (SD) age of 13.6 (0.3), 33% (*n* = 969) of the participants experienced dental caries, of whom 764 children experienced mild caries (DMFT 1-3) and 205 children experienced severe caries (DMFT >3). The mean (SD) value of caries lesions in the population having mild dental caries was 1.7 (0.8), and for severe caries, this was 5.3 (1.8) (table 1).

**Table 1:** Characteristics of the total study population

	Total population (*n* = 2911)	No caries (*n* = 1942)	Mild caries (*n* = 764)	Severe caries (*n* = 205)
Gender				
Boys	1403 (48.2%)	981 (50.5%)	338 (44.2%)	84 (41.0%)
Girls	1508 (51.8%)	961 (49.5%)	426 (55.8%)	121 (59.0%)
Total energy intake (kcal/day)	1494 ± 375	1495 ± 363	1484 ± 382	1516 ± 452
Diet quality score	4.53 ± 1.22	4.6 ± 1.2	4.5 ± 1.2	4.3 ± 1.3
Age at dental assessment (year)	13.6 ± 0.3	13.6 ± 0.3	13.6 ± 0.4	13.7 ± 0.4
Dental caries experience	0.8 ± 1.6	NA	1.7 ± 0.8	5.3 ± 1.8
Brushing frequency (per day)				
≤Once	483 (17.9%)	300 (16.5%)	134 (19.2%)	49 (27.8%)
≥Twice	2208 (82.1%)	1518 (83.5%)	563 (80.8%)	127 (72.2%)
Missings	*7.6%*	*6.4%*	*8.8%*	*14.1%*
Other hygiene method than tooth brushing				
Yes	1263 (46.8%)	983 (53.9%)	333 (47.6%)	89 (50.3%)
No	1437 (53.2%)	841 (46.1%)	366 (52.4%)	88 (49.7%)
Missings	*7.2%*	*6.1%*	*8.5%*	*13.7%*
Maternal educational level				
Low	205 (7.4%)	106 (5.7%)	70 (9.7%)	29 (15.7%)
Middle	732 (26.4%)	469 (25.1%)	194 (26.9%)	69 (37.3%)
High	1834 (66.2%)	1291 (69.2%)	456 (63.3%)	87 (47.0%)
Missings	*4.8%*	*3.9%*	*5.8%*	*9.8%*
Net household income per month				
Low (<€3600)	933 (36.9%)	562 (32.8%)	277 (42.2%)	94 (59.9%)
High (≥€3600)	1597 (63.1%)	1154 (67.2%)	380 (57.8%)	63 (40.1%)
Missings	*13.1%*	*11.6%*	*14.0%*	*23.4%*
Ethnic background				
Dutch	2004 (69.2%)	1399 (72.2%)	497 (65.8%)	108 (53.2%)
Non-Dutch	891 (30.8%)	538 (27.8%)	258 (34.2%)	95 (46.8%)
Missings	*0.5%*	*0.3%*	*1.2%*	*1.0%*

Notes: Values are means ± SD for continuous variables, and absolute numbers with valid percentages for categorical variables. The percentages of missing information of covariates are indicated in *italic* type.

The results of the multinomial regression models showed that one unit increase in the diet quality score was associated with lower odds of severe dental caries after adjustments for age, gender and total energy intake (model 1: OR 0.80, 95% CI 0.70–0.90), and after adjustments for socioeconomic indicators and ethnic background (model 2: OR 0.88, 95% CI 0.77–0.99). After adjustment for oral hygiene practices, this estimate remained similar but was no longer statistically significant (model 3: OR 0.89, 95% CI 0.78–1.01). In line with this, children in the highest quartile (quartile 4) of the diet quality score showed a lower occurrence of severe dental caries in models 1 (OR 0.45, 95% CI 0.31–0.75) and model 2 (OR 0.62, 95% CI 0.39–0.98), which was no longer statistically significant in model 3 (OR 0.65, 95% CI 0.41–1.03) (table 2). However, there was no systematic trend for the association of higher diet quality scores with lower dmft scores (0.056 < *P*_trend_ < 0.742), except for model 1 (*P*_trend_ < 0.001).

**Table 2:** Associations between diet quality and dental caries using multinomial regression models

	Model 1		Model 2		Model 3	
	Mild dental caries	Severe dental caries	Mild dental caries	Severe dental caries	Mild dental caries	Severe dental caries
Diet quality score	0.97 (0.90–1.05)	0.80 (0.70–0.90)	1.01 (0.93–1.08)	0.88 (0.77–0.99)	1.01 (0.94–1.09)	0.89 (0.78–1.01)
Quartiles of diet quality score						
Quartile 1 (lowest adherence)	Ref	Ref	Ref	Ref	Ref	Ref
Quartile 2	0.84 (0.66–1.06)	0.77 (0.53–1.14)	0.87 (00.68–1.11)	0.84 (0.57–1.25)	0.87 (0.69–1.12)	0.86 (0.58–1.28)
Quartile 3	0.93 (0.73–1.18)	0.69 (0.46–1.03)	0.99 (0.78–1.27)	0.82 (0.54–1.25)	1.00 (0.79–1.28)	0.87 (0.57–1.32)
Quartile 4 (highest adherence)	0.91 (0.72–1.17)	0.48 (0.31–0.75)*	1.00 (0.78–1.29)	0.62 (0.39–0.98)*	1.01 (0.79–1.30)	0.65 (0.41–1.03)
*P*_trend_	0.647	0.001*	0.742	0.056	0.669	0.093

Notes: Effect estimates represent ORs with 95% CIs for the association between diet quality (per one point or in quartiles with the lowest quartile as reference) with mild (*n* = 764) or severe caries (*n* = 205) using caries-free children (*n* = 1942) as reference estimated using multinomial regression models. Model 1: adjusted for age at dental assessment, gender and total energy intake; Model 2: model 1 + additional adjustments for ethnic background, educational level of mother, net family household income; Model 3: model 2 + additional adjustments for brushing frequency and additional oral hygiene methods.

* Statistical significance.

Omitting components from the diet quality score one by one did not change the association between the diet quality score and dental caries (Supplementary table S1). Children in the highest quartile (quartile 4) of the diet quality score and with poor oral hygiene showed a lower occurrence of severe dental caries in models 1 and 2 (model 1: OR 0.35, 95% CI 0.13–0.90; model 2: OR 0.42, 95% CI 0.16–1.13) than children with good oral hygiene (model 1: OR 0.59, 95% CI 0.35–1.02; model 2: OR 0.78, 95% CI 0.45–1.34) (Supplementary table S2).

## Discussion

### Main findings

In our study population, adherence to dietary guidelines was on average low, with a mean diet quality score of 4.5 out of 10 points, and two-third of the study population had a caries-free dentition. The results of the regression analysis between diet quality and dental caries showed that better adherence to dietary guidelines is associated with decreased dental caries. However, after adjustments for oral hygiene practices, this association became only tentatively significant.

### Explanation of findings and comparison with previous literature

The observed association between better diet quality and lower occurrence of dental caries could potentially be attributable to a reduction in sugar intake. That was also assumed in the development of the Dutch nutritional guidelines, although the components that formulate the Dutch dietary guidelines contain no specific guidance on sugar intake.^10^ This theory is supported by the observation that the added sugar intake in grams per day was slightly lower among children with the highest adherence to the dietary guidelines compared with the other children (Supplementary table S3). Sugar-containing beverages might be therefore attributable to the observed relationship in this study, but omitting this component from the total score did not change the effect size of the present association (Supplementary table S1). It has been indicated earlier that higher diet quality scores are related to higher frequency and regularity of eating.^25^ Given that the development of dental caries is based on the imbalance between demineralization and remineralization of the enamel, the frequency of nutrition intake could also be an explanation for the reduced occurrence of dental caries among those with better diet.^8^^,^^26–28^ Namely that the maximum number of moments of food intake per day is lower among children with higher diet quality scores. Moreover, the composition of the diet could also be an explanation for the observed results. Previous research showed that a diet high in starch leads to lower levels of dental caries.^3^ One of the components of the diet quality score is fruit intake. Fruit also contains sugar, in the form of fructose. But there is limited evidence available that normal intake of fruits could play a role in the development of dental caries.^29^ Various previous studies indicated that fruits are less cariogenic than sucrose.^29^ Also, a diet rich in calcium, phosphate and protein might favour remineralization and could thereby decrease the risk of caries.^30^ Milk products are included in the dietary guidelines and contain lactose as a carbohydrate, which is the least cariogenic sugar, and high levels of calcium, phosphate and casein, which may therefore favour oral health.^29^^,^^31^

The findings of our study are in line with those from the limited number of previous studies that investigated adherence to dietary guidelines and dental caries. A previous study from the USA showed that children with a higher diet quality (highest tertile), expressed as the HEI which is based on the US food pyramid, had a statistically significantly lower risk to develop early childhood caries than children with low adherence to the food pyramid (lowest tertile).^11^ The researchers adjusted for sociodemographic characteristics, but not for oral hygiene practices. Another small cross-sectional study from Egypt showed in a univariate analysis that an increase in the HEI was associated with lower levels of early childhood caries and severe early childhood caries.^32^ The association of better diet quality on lower risk of dental caries has also been shown among adults from the USA.^12^ However, the association was only observed in foreign-born participants, and the study’s authors speculate that this might be due to the long-term exposure to fluoride, through water or toothpaste, that US-born participants have had.^12^

Fluoride exposure could be an explanation for the observed small attenuation in effect estimates towards non-significance after adjustments for oral hygiene methods in our study. Namely, it is known that remineralization of the enamel is enhanced through fluoride exposure. In the Netherlands, most individuals use a toothpaste containing fluoride, and in our study population, 82.1% had a brushing frequency of twice or more per day. When stratifying the association between diet quality and dental caries for brushing frequency, a lower odds of severe dental caries was observed in the presence of better diet quality among children who brushed their teeth once or less per day than those who brushed twice or more per day (Supplementary table S2). It is well established in previous literature that the relationship between carbohydrate consumption and caries is weaker in the presence of fluoride exposure.^33^^,^^34^

### Strengths and weaknesses

The results of our study should be seen in the light of some limitations. This cohort study is clearly prone to high numbers of loss to follow-up and drop-outs. This has resulted in a smaller sample size, and thus a decreased power. This may also be an explanation for the statistical non-significance in model 3 due to wide CIs of the results where effect sizes clearly indicated an association. Besides, selection bias due to differential participation could also have played a role, since participants with missing data on diet quality or dental caries had lower diet quality scores, thus less adherences to dietary guidelines, but higher levels of dental caries (Supplementary table S4). This could have induced an underestimation of the observed associations. Furthermore, in the long time period between the assessment of diet quality at the age of 8 years and dental caries assessment at the age of 13 years, dietary patterns might be changed and be more influenced by the young adolescent itself.^35^^,^^36^ However, DMFT is a cumulative measure indicating caries activity from permanent dentition onwards and includes both treated and untreated caries in the permanent teeth. Moreover, in our study, the diet quality score was assessed by means of an FFQ. FFQs are prone to measurement errors, but they are commonly used in nutritional epidemiological studies to rank participants according to their dietary intake.^37^ The FFQ used in this study was validated against the doubly labelled water method and showed moderate to good agreement.^14^ Though the dietary assessment based on an FFQ estimates the frequency and portion size information about food intake in the past month, no information regarding the daily food intake could be estimated based on the used FFQ. Since the frequency of eating is relevant in the development of dental caries,^26^^,^^27^ future studies are encouraged to assess diet using repeated 24-hour recalls in order to incorporate the frequency of food intake when studying the association between dietary intake and dental caries. Furthermore, since it is more difficult to differentiate between stages of caries development using intra-oral photographs, a potential underestimation of caries could have occurred. However, comparing the prevalence of dental caries to national statistics gives almost equal proportions, with approximately 38% of the 11 years aged Dutch population being affected by dental caries.^38^ Lastly, at the age of 13 years, some children might not have exfoliated some primary teeth. Some children therefore might be classified as caries-free based on the determination of DMFT in the permanent dentition, but in fact had caries in the primary dentition which might have been caused by nutritional practices. We partly took this into account by adjusting for age at the caries assessment in our main analyses. Nevertheless, this limitation could have led to a non-differential information bias, which could have diluted the effect estimates.

The major strength of the current study is the multi-ethnic cohort in which data were assessed longitudinally. Though there was a substantial loss to follow-up, the sample size was still considerably high. It is the first study on adherence to dietary guidelines and dental caries in a European setting. By studying the overall dietary intake, we also took into account the interaction between nutrients and foods, which is not possible when investigating individual nutritional components.^39^ Also, we were able to adjust for several important socioeconomic indicators and oral hygiene habits that could have confounded or moderated the relationship between adherence to dietary guidelines and dental caries in previous studies.

## Conclusion and implications

We have shown that adherence to the national Dutch dietary guidelines has the potential to reduce dental caries in young adolescents, since it was associated with less dental caries lesions. However, in the presence of proper oral hygiene, this association is attenuated. Although the total amount of dietary intake may be important in the development of dental caries, future studies on diet and dental caries are warranted in larger datasets and researchers are encouraged to include the frequency of eating to better understand the role of dietary patterns and dental caries.

## Supplementary Material

ckad097_Supplementary_Data

## Data Availability

The data that support the findings of this study are available on request from the corresponding author. The data are not publicly available due to privacy or ethical restrictions. Key points There is very limited research on the relation between an overall diet and dental caries. Within the study population dental caries was prevalent (33%), and adherence to dietary guidelines was poor. Higher diet quality scores were associated with lower levels of dental caries. Adherence to the Dutch dietary guidelines has the potential to reduce dental caries. In the presence of proper oral hygiene, the association is attenuated.
