# Supplementary material for: Adherence to dietary guidelines and dental caries among children: a longitudinal cohort study
Source: Eur J Public Health. 2023 Jun 22;33(4):653–8. doi: 10.1093/eurpub/ckad097 (PMC10393494; doi:10.1093/eurpub/ckad097)
Supplement: ckad097_Supplementary_Data [file ckad097_supplementary_data.docx]

# Electronic Supplementary material

**Article title:** Adherence to dietary guidelines and dental caries among children: a longitudinal cohort study
**Author names:** Agatha W. van Meijeren - van Lunteren a, b; Trudy Voortman c; Eppo B. Wolvius a, b; and Lea Kragt a, b
**Affiliations:** a The Generation R Study Group, Erasmus MC University Medical Center, PO Box 2040, 3000 CA Rotterdam, the Netherlands; b Department of Oral & Maxillofacial Surgery, Special Dental Care and Orthodontics, Erasmus MC University Medical Center, PO Box 2040, 3000 CA Rotterdam, the Netherlands; c Department of Epidemiology, Erasmus MC University Medical Center, PO Box 2040, 3000 CA Rotterdam, the Netherlands
**Content of this file:** This electronic supplementary material contains tables with the results of several supplementary analyses which support the results of the main manuscript.

**Supplementary Table 1 - Associations between variants of the continuous diet quality score and dental caries ^a^**

|  | **Model 1** |  | **Model 2** |  | **Model 3** |  |
| --- | --- | --- | --- | --- | --- | --- |
| Variants of diet quality score | **Mild dental caries**  **OR (95% CI)**^b^ | **Severe dental caries OR (95% CI)**^b^ | **Mild dental caries**  **OR (95% CI)**^b^ | **Severe dental caries**  **OR (95% CI)**^b^ | **Mild dental caries**  **OR (95% CI)**^b^ | **Severe dental caries**  **OR (95% CI)**^b^ |
| Total diet quality score | 0.97 (0.90-1.05) | **0.80 (0.70-0.90)** | 1.01 (0.93-1.08) | **0.88 (0.77-0.99)** | 1.01 (0.94-1.09) | 0.89 (0.78-1.01) |
| Diet quality score without fruit | 0.97 (0.90-1.05) | **0.79 (0.70-0.91)** | **1.00 (0.92-1.08)** | **0.86 (0.75-0.99)** | 1.00 (0.92-1.08) | 0.88 (0.77-1.01) |
| Diet quality score without vegetables | 0.97 (0.90-1.05) | **0.78 (0.69-0.90)** | **1.00 (0.92-1.08)** | **0.85 (0.74-0.97)** | 1.01 (0.93-1.09) | **0.87 (0.75-0.99)** |
| Diet quality score without grains | 1.01 (0.93-1.09) | **0.85 (0.74-0.97)** | 1.03 (0.95-1.12) | 0.91 (0.79-1.04) | 1.04 (0.96-1.12) | 0.93 (0.81-1.06) |
| Diet quality score without fish | 1.00 (0.92-1.09) | **0.79 (0.69-0.91)** | 1.04 (0.96-1.13) | 0.89 (0.77-1.02) | 1.04 (0.96-1.14) | 0.90 (0.78-1.04) |
| Diet quality score without legumes | 0.93 (0.86-1.01) | **0.71 (0.62-0.81)** | 0.98 (0.90-1.07) | **0.82 (0.71-0.94)** | 0.99 (0.91-1.07) | **0.83 (0.73-0.96)** |
| Diet quality score without nuts | 0.96 (0.89-1.04) | **0.76 (0.67-0.87)** | 0.99 (0.92-1.08) | **0.84 (0.73-0.96)** | 1.00 (0.92-1.08) | **0.86 (0.75-0.98)** |
| Diet quality score without dairy | 0.99 (0.91-1.07) | **0.84 (0.73-0.96)** | 1.01 (0.94-1.10) | 0.90 (0.79-1.03) | 1.02 (0.94-1.10) | 0.92 (0.80-1.06) |
| Diet quality score without fats | 0.97 (0.90-1.05) | **0.82 (0.72-0.94)** | 1.00 (0.93-1.08) | 0.89 (0.78-1.02) | 1.01 (0.93-1.09) | 0.91 (0.80-1.04) |
| Diet quality score without sugar-containing beverages | 0.97 (0.90-1.04) | **0.79 (0.70-0.90)** | 1.01 (0.93-1.09) | **0.88 (0.77-1.00)** | 1.01 (0.93-1.09) | 0.90 (0.79-1.02) |
| Diet quality score without meat | 0.96 (0.89-1.03) | **0.77 (0.68-0.88)** | 0.99 (0.92-1.07) | **0.86 (0.76-0.98)** | 1.00 (0.92-1.08) | 0.88 (0.77-1.00) |

Model 1: adjusted for age at dental assessment, gender, and total energy intake.

Model 2: model 1+ additional adjustments for ethnic background, educational level of mother, net family household income

Model 3: model 2+ additional adjustments for brushing frequency and additional oral hygiene methods

^a^ Effect estimates were estimated using multinomial regression models.

^b^ Effect estimates represent odds ratios (OR) with 95% confidence intervals (95% CI) for the association between mild or severe caries using caries free children as reference.

**Supplementary Table 2 - Associations between diet quality and dental caries using multinomial regression models, stratified for brushing frequency**

|  | Model 1 | | Model 2 | |
| --- | --- | --- | --- | --- |
| Brushes once or less per day | **Mild dental caries** | **Severe dental caries** | **Mild dental caries** | **Severe dental caries** |
| Diet quality score | 0.90 (0.76-1.07) | **0.73 (0.58-0.93)** | 0.94 (0.78-1.12) | 0.80 (0.62-1.03) |
| Quartiles of diet quality score |  |  |  |  |
| Quartile 1 (lowest adherence) | Ref | Ref | Ref | Ref |
| Quartile 2 | 0.86 (0.50-1.48) | 0.78 (0.35-1.71) | 0.92 (0.53-1.59) | 0.79 (0.35-1.76) |
| Quartile 3 | 0.56 (0.30-1.06) | **0.44 (0.19-1.00)** | 0.59 (0.31-1.14) | 0.55 (0.24-1.30) |
| Quartile 4 (highest adherence) | 0.88 (0.79-1.57) | **0.35 (0.13-0.90)** | 0.97 (0.53-1.76) | 0.42 (0.16-1.13) |
| Brushes twice or more per day | **Mild dental caries** | **Severe dental caries** | **Mild dental caries** | **Severe dental caries** |
| Diet quality score | 1.00 (0.92-1.09) | **0.85 (0.73-1.00)** | 1.03 (0.94-1.12) | 0.93 (0.80-1.09) |
| Quartiles of diet quality score |  |  |  |  |
| Quartile 1 (lowest adherence) | Ref | Ref | Ref | Ref |
| Quartile 2 | 0.85 (0.65-1.12) | 0.84 (0.51-1.38) | 0.88 (0.66-1.16) | 0.92 (0.55-1.53) |
| Quartile 3 | 1.05 (0.80-1.39) | 0.86 (0.53-1.41) | 1.12 (0.84-1.48) | 1.01 (0.61-1.67) |
| Quartile 4 (highest adherence) | 0.96 (0.72-1.26) | 0.59 (0.35-1.02) | 1.03 (0.78-1.37) | 0.78 (0.45-1.34) |

Effect estimates represent odds ratios (OR) with 95% confidence intervals (95% CI) for the association between diet quality (per 1 point or in quartiles with the lowest quartile as reference) with mild or severe caries using caries free children as reference estimated using multinomial regression models.

Model 1: adjusted for age at dental assessment, gender, and total energy intake.

Model 2: model 1+ additional adjustments for ethnic background, educational level of mother, net family household income

**Supplementary Table 3 –Added sugar intake at the age of 8 years per quartile of the diet quality score**

|  | Added sugar intake g/d |
| --- | --- |
| **Quartiles of diet quality score** |  |
| Quartile 1 (lowest adherence) | 62.5 ± 36.7 |
| Quartile 2 | 59.5 ± 30.3 |
| Quartile 3 | 58.6 ± 31.7 |
| Quartile 4 (highest adherence) | 53.3 ± 28.8 |

Numbers are presented as mean ± SD

Supplementary Table 4 - Non-response analysis comparing characteristics of the study population with the non-eligible population due to missing data on diet quality and/or dental caries at follow-up (> 8 year)

|  | Included (n=2,911) | Excluded (n=4,751) |
| --- | --- | --- |
| Child’s gender |  |  |
| Boys | 1,403 (48.2%) | 2,449 (51.6%) |
| Girls | *1,508 (51.8%)* | 2,301 (48.41%) |
| *Missings* | *NA* | *0.02%* |
| Childs age at dietary intake assessment | 8.1 ± 0.2 | 8.2 ± 0.3 |
| *Missings* | *NA* | *60.6%* |
| Total energy intake (kcal/d) | 1,493.8 ± 375.0 | 1,486.8 ± 406.5 |
| *Missings* | *NA* | *61.7%* |
| Diet quality score | 4.53 ± 1.22 | 4.47 ± 1.23 |
| *Missings* | *NA* | *61.7%* |
| Child’s age at dental assessment | 13.6 ± 0.3 | 13.7 ± 0.4 |
| *Missings* | *NA* | *61.0%* |
| Dental caries, mean ± SD | 0.8 ± 1.6 | 1.5 ± 2.4 |
| *Missings* | *NA* | *78.2%* |
| Dental caries in categories, n (%) |  |  |
| No caries (DMFT 0) | 1,942 (66.7%) | 559 (54.0%) |
| Mild caries (DMFT 1-3) | 764 (26.2%) | 310 (29.9%) |
| Severe caries (DMFT >3) | 205 (7.0%) | 167 (16.1%) |
| *Missings* | *NA* | *78.2%* |
| Brushing frequency (p/d) |  |  |
| ≤ Once | 483 (17.9%) | 395 (22.2%) |
| ≥ Twice | 2,208 (82.1%) | 1,386 (77.8%) |
| *Missings* | *7.6%* | *62.5%* |
| Other hygiene method than tooth brushing |  |  |
| Yes | 1,263 (46.8%) | 858 (47.7%) |
| No | 1,437 (53.2%) | 939 (52.3%) |
| *Missings* | *7.2%* | *62.2%* |
| Maternal educational level |  |  |
| Low | 205 (7.4%) | 566 (16.7%) |
| Middle | 732 (26.4%) | 1,146 (33.8%) |
| High | 1,834 (66.2%) | 1,675 (49.5%) |
| *Missings* | *4.8%* | *28.7%* |
| Net income per month |  |  |
| Low (< €3600) | 933 (36.9%) | 791 (47.8%) |
| High (≥ €3600) | 1,597 (63.1%) | 864 (52.2%) |
| *Missings* | *13.1%* | *65.2%* |
| Ethnic background |  |  |
| Dutch | 2,004 (69.2%) | 2,286 (50.5%) |
| Non-Dutch | 891 (30.8%) | 2,238 (49.5%) |
| *Missings* | *0.5%* | *4.8%* |

Values are means ± SD for continuous variables, and absolute numbers with valid percentages for categorical variables. The percentages of missing information of covariates are indicated in *italic* type.
